# Numerical study on inter-particle effects for multiple reacting biomass and coal particles based on Micro-CT morphology

**DOI:** 10.1016/j.heliyon.2024.e40419

**Published:** 2024-11-15

**Authors:** Dongyu Liang

**Affiliations:** A. Leon Linton Department of Mechanical, Robotics and Industrial Engineering, Lawrence Technological University, Southfield, MI, 48075, United States

**Keywords:** Coal, Biomass, Co-firing, Morphology, Group effects

## Abstract

Co-firing of biomass with coal combines the environmental benefits of renewable biomass with the high energy content of coal. Although the common numerical simulation treats the biomass and coal particles with ideal morphology, real particles often demonstrate nonsmoothed surface and irregular shape. To understand the impact of particle morphology in a group of biomass and coal particles co-firing together and to inform simple models appropriate, this study investigated the interparticle effects among particles using realistic particle morphology, focusing on fluid dynamics such as temperature distribution, flow patterns and drag coefficients. Particle-scale computational fluid dynamics (CFD) simulations using micro-CT imaging showed that realistic particle shapes resulted in nonuniform flow fields and temperature distributions with different reaction intensities due to the species transportation. It is in contrast to traditional ideal shape models, which often rely on simplified spherical representations of particles and cannot capture the intricacies of real particle shapes. Realistic models revealed more complex surfaces, highly irregular particle structures, and varied reaction zones that affected the overall dynamics. In addition, changing the orientation of one particle affects the combustion characteristics of neighboring particles. This effect is also not captured while using the spherical structure. These differences underscore the critical impact of particle morphology on drag and heat transfer, thereby challenging conventional spherical models. The findings of this study advocate for a paradigm shift in CFD modeling approaches, emphasizing the importance of realistic particle representation to improve the accuracy of predictions and enhance the co-firing system efficiency.

## Highlights

• The new CFD approach integrates micro-CT for realistic particle morphology in co-firing.
• Revealed significant impact of real particle shapes on combustion and gasification.
• Comparative analysis shows inequalities with traditional spherical particle models.
• Findings encourage for a paradigm shift in modeling for enhanced energy efficiency.
• Study underscores importance of particle interactions in optimizing co-firing reactors.

## 1 Introduction

The pursuit of sustainable and efficient energy production has resulted in studies focusing on the co-firing of biomass and coal, an approach that synergizes the environmental benefits of biomass with the high energy content of coal [1]. Co-firing, the process of burning biomass together with coal, offers a pragmatic solution for reducing greenhouse gas emissions and reliance on fossil fuels. Biomass, a renewable energy source, has a considerably lower carbon footprint than coal, and its incorporation into existing coal-fired power plants can significantly reduce the overall carbon dioxide emissions [2,3]. This method leverages existing infrastructure and aids in the gradual transition to more sustainable energy sources. Current research on biomass and coal co-combustion has primarily focused on simplified particle models, often using spherical representations with shape factors [[4], [5], [6], [7]]that do not capture the complexities of real particle shapes. However, numerical studies focusing on interparticle interactions between coal and biomass particles remain limited, indicating a significant gap in our understanding of multiparticle dynamics in co-firing systems.

Studying co-firing at the particle scale is important for resolving the complexities of this thermochemical process. At the particle scale, each particle acts as a microreactor where a multitude of physical and chemical phenomena, ranging from heat and mass transfer to chemical reactions, unfold [8]. The behavior of individual particles can significantly affect the reactor-scale output, such as carbon conversion and temperature[[9], [10], [11]]. Strategies to modify reactor design and improve operating conditions necessitate a more comprehensive understanding of particle-scale phenomena. However, the submodels used in common reactor-scale studies for co-firing biomass with coal are still based on the assumption of a spherical char particle structure [12,13]. This simplification limits the accuracy of simulations in predicting real-world behavior, particularly when considering the irregular shapes of actual biomass and coal particles. The significance of the particle-scale study has been further amplified by considering interparticle effects among multiple char particles [14,15]. These interactions significantly influence the overall reaction dynamics. For instance, the proximity of particles can affect local temperature gradients, gas concentrations, and reaction rates, resulting in nonuniform reaction zones. Thermal radiation exchange between particles and the modification of local flow fields are among the critical interparticle phenomena that can affect carbon conversion [16]. The clustering of particles and formation of char bridges can alter the permeability of the reactor and gas flow paths, further complicating the process [17].

In this case, the efficacy of co-firing biomass with coal depends on a deeper understanding of interparticle interactions during the combustion and gasification processes. However, the application of experiments to study interparticle interactions at the particle scale is normally challenging and requires a laboratory reactor with a high-speed camera, laser, and deflector [15,18]. Compared to actual experiments with a critical environment that is difficult to measure or observe, computational fluid dynamics (CFD) simulations have emerged as a powerful tool in this context. They offer detailed insights at the particle scale and present unparalleled insights into the complex interplay of physical and chemical phenomena that govern this process [19,20]. However, numerical studies focusing on interparticle interactions between coal and biomass particles are limited. Another significant limitation of the current scope of CFD applications is the predominant focus on single-particle models[[21], [22], [23], [24], [25]]. While these studies offer valuable insights into the fundamental reactions and transformations that occur during gasification and combustion, they fail to capture the collective dynamics and interparticle effects that are intrinsic to multiparticle systems found in practical reactors.

The interaction between particles is a complex phenomenon influenced by various factors such as particle size, shape, density, and chemical composition. These interactions can significantly affect the combustion and gasification characteristics, including the burnout rate, heat release, and emission profiles [14,26]. Moreover, these effects would be more substantial for co-firing systems, wherein the morphologies of biomass and coal vary [27,28]. Despite advancements in CFD modeling and its application in studying co-firing, current research often falls short owing to the oversimplification of particle structures[14,[29], [30], [31]]. Most studies have employed idealized geometries, typically spherical, to represent biomass and coal particles. In single-particle studies, considerable efforts have been made to include complex particle shapes. Discrete networks have been used to model char consumption. However, their ability [32] to emulate real char structures and integrate them with other physics is limited. Confocal scanning laser microscopy was used to generate an idealized biomass structure with morphological parameters for nonreacting simulations only [33]. A larger sphere comprising over hundred smaller monodisperse nonporous spheres was also modeled for oxy-combustion [34]. A new approach for particle structure was developed to study group effects by overlapping multiple spherical particles [35], indicating the importance of particle shape factors in CFD–DEM simulations. However, the overall complexity of the actual shapes of the biomass and coal particles remains unclear. These approaches overlook the inherent irregularities and complex shapes of real particles. The importance of realistic morphology has been demonstrated in single-particle research; however [36], it has not yet been well discussed for interparticle effects in a group of particles.

To address all the gaps caused by the idealized particle structure, this study innovatively used micro-computed tomography (micro-CT) techniques [8,27] to capture realistic biomass and coal particle morphologies. Further, to overcome the single particle limitation, multiple particles reconstructed from the micro-CT were integrated into CFD simulations to explore inter-particle effects during co-firing. Micro-CT is an advanced, nondestructive method for capturing the intricate shapes and structures of particles at high resolution [37]. X-ray CT has recently been used to study the porosity distribution and reconstruct the internal and external structures of both biomass and coal particles[[38], [39], [40]]. This level of detail is crucial because the physical characteristics of char particles significantly influence the kinetics of reactions, heat and mass transfer processes, and overall efficiency as indicated in single particle combustion research. As preliminary research working on the group effects, this study first focus on the effect of realistic particle shapes on the fluid dynamics among multiple particles and comparing parameters such as velocity, temperature, and species distribution from simulations using both real and idealized particle structures. The proposed study is the first step which can be built upon in future studies that incorporate more complex mechanisms. In later research, the following work aims to bridge the gap in the current understanding and contribute significantly to the optimization of co-firing systems. This comparative analysis provides essential data for improving CFD models, resulting in more efficient co-firing practices.

## 2 Methods

### 2.1 Biomass and coal char particles

To generate char particles with varied shapes, both coal (Illinois #6) and biomass (Pine sawdust from Larrabee, Iowa) samples were sifted to achieve a uniform diameter of approximately 100 μm. These particles were then evenly dispersed on an aluminum foil sheet enveloped in argon atmosphere. The setup was then placed in a beaker to ensure a tight seal before being inverted and placed in a furnace. The furnace conditions were meticulously controlled, with the temperature set to 1073 K, and the samples were subjected to a rapid heating phase, achieving a remarkable rate of approximately 10000 K/s within a brief duration of 30 s. For this study, we selected two particle shapes of bituminous coal, Illinois #6 with significant different aspect ratios and a particular biomass variety, pine sawdust, sourced from a woodworking facility in Larrabee, Iowa, United States of America. These materials were chosen with precision to facilitate a focused examination of the interparticle dynamics between single coal and single biomass particles, and then later expanded to three particles to investigate their intricate interactions during the combustion processes.

### 2.2 Image processing and meshing

**Fig. 1:**
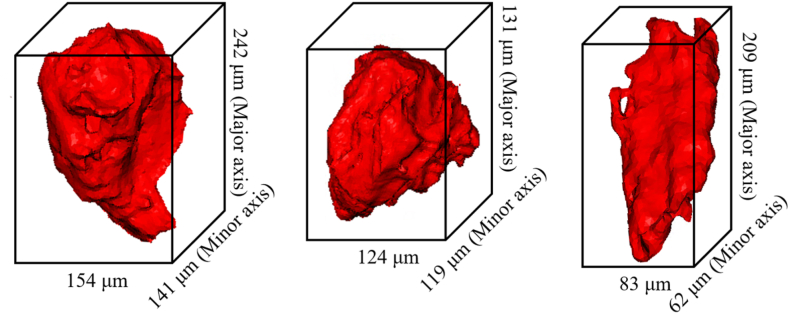
3D coal (left and middle) and biomass (right) particles.

Numerous coal and biomass char particles, which were meticulously prepared earlier, were imaged using a micro-CT scanner. The operating conditions for this imaging process are outlined in detail in Ref. [22]. Following imaging, a comprehensive stack of TIFF images was imported into ScanIP. This platform facilitates segmentation and structure generation by setting a stage for subsequent meshing. To distinguish the char particles from their surroundings, a thresholding technique was employed to isolate them from the background. As illustrated in Fig. 1, the reconstructed three-dimensional (3D) models of the particles revealed highly irregular contours marked by surface protrusions that added to their complexity. Notably, biomass particles are characterized by a significantly higher aspect ratio, underscoring the distinct morphological differences between coal and biomass char particles.

**Fig. 2:**
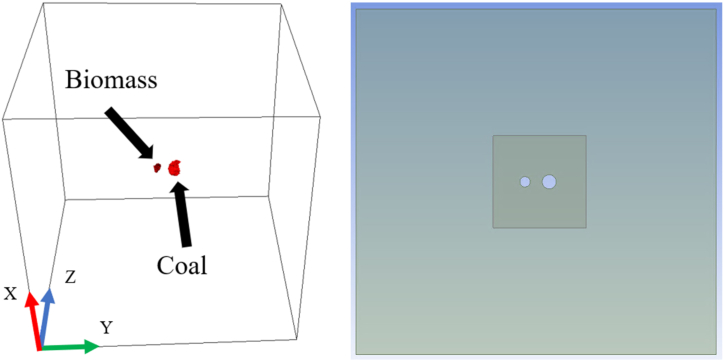
3D (left) and 2D (right) computational domains.

As a demonstration, Fig. 2 (a) shows two particles, biomass(left) and coal (right), placed at the center of a cubic domain with a length of 3 mm. The distance (L) between two particles was half the average major axis length, which can be changed later. The same strategy was subsequently applied to the three-particle simulation. To bridge the gap between the complex reality of particle shapes and the simplified models commonly used in computational studies, Fig. 2 (b) shows a two-dimensional (2D) computational domain with two spherical particles placed at the center. To ensure a valid comparison, the radius of each spherical particle was based on the effective radius (m) calculated from the 3D morphology using

(1) $$\begin{array}{c} R_{p}=\sqrt[{3}]{\frac{3V}{4\pi }} \end{array}$$

where V is the particle volume ($m^{3}$) measured from the 3D geometries obtained from the micro-CT images. To accurately capture the intricacies of the uneven surfaces of the particles and ensure the generation of a high-quality mesh, a smaller cubic domain was positioned. This strategic setup facilitated gradual refinement of the mesh, transitioning from coarser elements in areas further away to finer ones closer to the particle, particularly near the critical gas boundary layer surrounding it. This method ensured that the mesh density was optimized for both the representation of the particle's complex surface features and the computational efficiency of the simulation. Following a mesh convergence study to determine the optimal balance between accuracy and computational resource demands, simulations were conducted using approximately four million mesh elements.

### 2.3 Numerical simulations

The 2D and 3D numerical simulation approaches are described in this section. The standard conservation equations for mass, momentum, species, and energy were solved in the boundary layer surrounding the particle in the domain as follows [41].

(2) $$\begin{array}{c} \frac{\partial \rho }{\partial t}+\nabla \left(\rho \vec{v}\right)=S_{m} \end{array}$$

(3) $$\begin{array}{c} \frac{\partial \rho \vec{v}}{\partial t}+\nabla \left(\rho \vec{v}\vec{v}\right)=-\nabla P+\nabla \left(\overset{═}{\tau }\right) \end{array}$$

(4) $$\begin{array}{c} \frac{\partial \rho Y_{i}}{\partial t}+\nabla \left(\rho \vec{v}Y_{i}\right)=-\nabla {\vec{J}}_{i}+R_{i} \end{array}$$

(5) $$\begin{array}{c} \frac{\partial \left(\rho h\right)}{\partial t}+\nabla *\left(\vec{v}\rho h\right)=\nabla \left(k\nabla T-\left(\sum_{i}h_{i}{\vec{J}}_{i}\right)+\left(\overset{═}{\tau }*\vec{v}\right)\right)+S_{f}^{h} \end{array}$$

where ρ is the density ($\frac{kg}{m^{3}}$), $\vec{v}$ is the velocity ($\frac{m}{s}$), $S_{m}$ is the mass source term ($\frac{kg}{m^{3}s}$), t is the time, P is the pressure (Pa), $\overset{═}{\tau }$ is the stress tensor (Pa), $Y_{i}$ is the mass fraction for species i, ${\vec{J}}_{i}$ is the diffusion flux for species i ($\frac{kg}{m^{3}s}$), $R_{i}$ is the rate of production ($\frac{kg}{m^{3}s}$) for species i, h is the enthalpy ($\frac{J}{kg}$), k is the thermal conductivity ($\frac{W}{mK}$) of the mixture, T is the temperature (K), and $S_{f}^{h}$ is the fluid enthalpy source term caused by reaction ($\frac{J}{m^{3}s}$). The ideal-gas law was used. Viscosity and thermal conductivity were calculated using kinetic theory and standard mixing rules. In this study, boundary conditions at a very distant location (1173 K and mole fractions of 23 % $O_{2}$ and 77 % $N_{2}$) were applied. The heterogeneous reaction mechanism of char conversion and homogeneous reaction of CO oxidation were considered in the simulation based on [42,43]. The Arrhenius equation was used for the reaction constant (r) calculation.

(6) $$\begin{array}{c} r_{i}=A_{i}\;\exp\left(\frac{-E_{a,i}}{R_{u}T_{p}}\right) \end{array}$$

where $A_{i}$ is the pre-exponential factor, $E_{a,i}$ is the reaction activation energy, $R_{u}$ is the gas constant. The kinetic parameters of the chemical reactions are listed in Table 1 [42].

**Table 1:** Kinetic parameters of chemical reactions.

Reactions	A (pre-exponential factor)	$E_{a}$ (J/mol)
R1 $2C+O_{2}\to 2CO$	1.97e+7	1.98e+5
R2 $C+{CO}_{2}\to 2CO$	1.29e+5	1.91e+5
R3 $2CO+O_{2}\to 2{CO}_{2}$	2.24e+12	1.67e+5

Further, we assumed the use of the pseudo-steady-state assumption because the transient burning of char particles can be well represented by a series of steady solutions at different levels of char conversion [44]. Steady-state simulations provide accurate solutions for a given morphology [27]. Hence, steady-state simulations were performed in ANSYS Fluent 2021R1 using a pressure-based solver and a coupled algorithm. The second-order upwind and PRESTO! schemes were used for the discretization. The particles were fixed in the flow field with a constant particle Reynolds number of 10 for all simulations. The particle Reynolds number is expressed as

(7) $$\begin{array}{c} Re=\frac{u2\bar{R_{p}}}{\upsilon } \end{array}$$

where u and υ are the velocity and kinematic viscosity of the incoming fluid, respectively, and $R_{p}$ is the effective particle radius averaged from coal and biomass.

To focus on the impact of particle morphology on interparticle effects while using ideal and realistic structures, all heterogeneous reactions with the same kinetic parameters were applied to the particle surface for all coal and biomass particles. Reactions within the solid particles and more detailed homogeneous and heterogeneous reactions will be introduced in later studies on coal and biomass.

## 3 Results and discussion

**Fig. 3:**
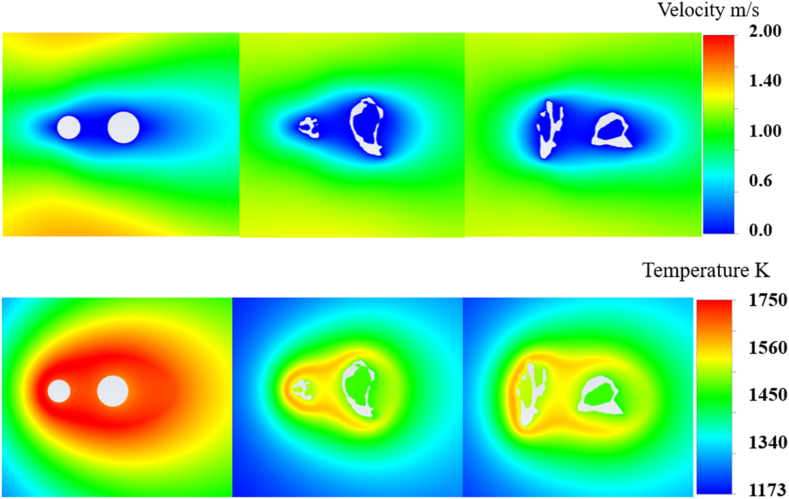
Velocity (top) and temperature (bottom) distributions from 2D (left) and 3D simulations with XY (middle) and YZ (right) plans.

Compared with the commonly used idealized spherical particle shape, this study aimed to explore the significance of incorporating real particle morphologies when examining interparticle effects for later research. In this case, the velocity and temperature distributions shown in Fig. 3 from both the 2D and 3D simulations provided comparisons of the heat transfer and fluid dynamics around the char particles. In the 2D simulation, similar to the temperature distribution, there was a smooth symmetric flow around the spherical particles. The expected streamlined behavior was observed with a clear velocity gradient as the flow accelerated around the particles. By contrast, 3D simulations with real particle morphologies exhibited more complex flow patterns. The irregular particle shapes disrupted the flow, creating regions of higher and lower velocities and temperatures that were not observed in the 2D simulation. Further, the 2D average surface temperature for the front particle was 1690K and 1668K for the rear particle. And the surface temperature dropped to 1539K and 1500K in 3D simulation. The overall temperature in the 3D simulation was lower than that in the 2D simulation, particularly downstream and at the location where the particle pore structure was significant. For 2D simulations, a uniform shape can result in a more consistent reaction surface, facilitating a more complete and quicker reaction process, which results in higher temperatures. In contrast to spherical particles, which have complicated uneven surfaces, pore structures, and enforced velocities, species such as O_2_ are difficult to transfer into the downstream area and pores from the surroundings, leading to a different reaction intensity and, therefore, a temperature level against the ideal structure. Based on the velocity and temperature figures, they both suggested that the real morphology facilitated non-uniform flow fields, which could have significant implications for the reaction rates and heat transfer in actual combustion processes. The differences in the flow patterns between the XY and YZ planes further emphasized the three-dimensional nature of these effects and the importance of using 3D models for accurate predictions.

**Fig. 4:**
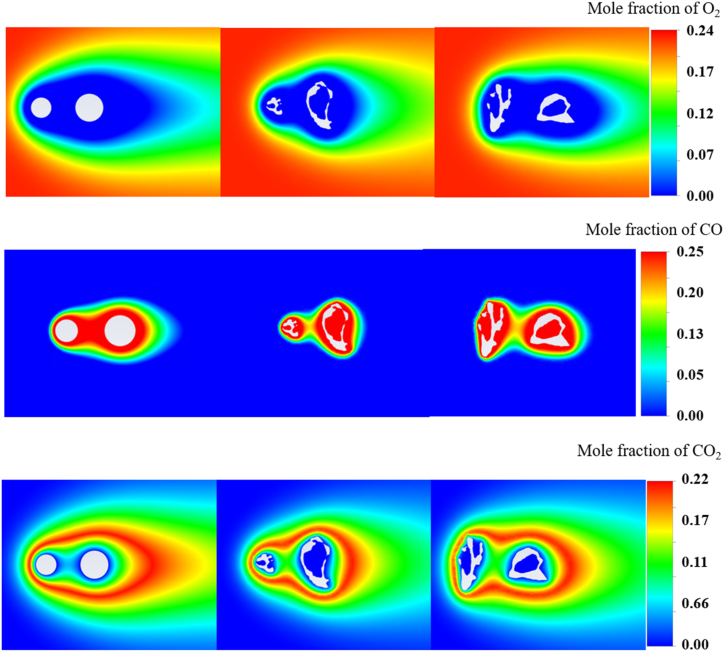
O_2_ (top), CO (middle) and CO_2_ (bottom) distributions from 2D and 3D simulations with XY and YZ plans.

Furthermore, the distributions of O_2_, CO, and CO_2_, as shown in Fig. 4, provided insightful data on the reaction process under the specific conditions of the study. The top panel illustrates the mole fraction of O_2_, the middle panel illustrates that of CO, and the bottom panel illustrates that of CO_2_, capturing the behavior of the chemical species in both the 2D and 3D simulations. Owing to the highly asymmetric 3D coal and biomass structures, the species distributions from two cross-sections across the XY (center) and YZ (right) planes are also provided.

In the O_2_ distribution, the presence of oxygen decreased as it approached the particle surface. The average mole fractions measured from both particles were 1.87e-287 for 2D and 3.33e-12 for 3D at the particle surface, indicating a stronger active reaction process for 2D due to the carbon oxidation reaction. The 3D simulation revealed a more heterogeneous pattern of oxygen reduction around the real particle morphology compared to the 2D simulation with spherical particles. O_2_ was asymmetrically distributed not only between the 2D and 3D structures but also between the XY and YZ planes changed particle morphology. This heterogeneity could be caused by the increased surface area and complex surface features of the real particles, which affect reactions with O_2_. The CO distribution highlighted the areas where heterogeneous reactions occurred, with CO being an intermediate product of R1, R2, and R3. The distribution indicated a higher CO mole fraction between the 2D biomass and coal particles, which is opposite to the 3D results, indicating more intensive carbon conversion in the 2D simulation. In the 3D simulation, the CO concentrations around the real particle morphology were notably higher and more irregularly shaped, reflecting more complex pathways and possibly slower mixing with ambient oxygen, owing to the particle's intricate shape. For CO_2_, the bottom panels showed the homogeneous reaction that occurred around the particles. Because of R2 reaction, CO_2_ was intensively consumed while approaching the particle surface and within the particles in 3D, leading to a low mole fraction of CO_2_. The distribution here was also markedly different between the 2D and 3D simulations. The actual particle morphology appeared to promote a more concentrated and less uniform distribution of CO_2_, particularly in the YZ plane. This could be interpreted as a result of the nonuniform reaction surface provided by the real particles, leading to localized areas with higher reaction rates.

**Fig. 5:**
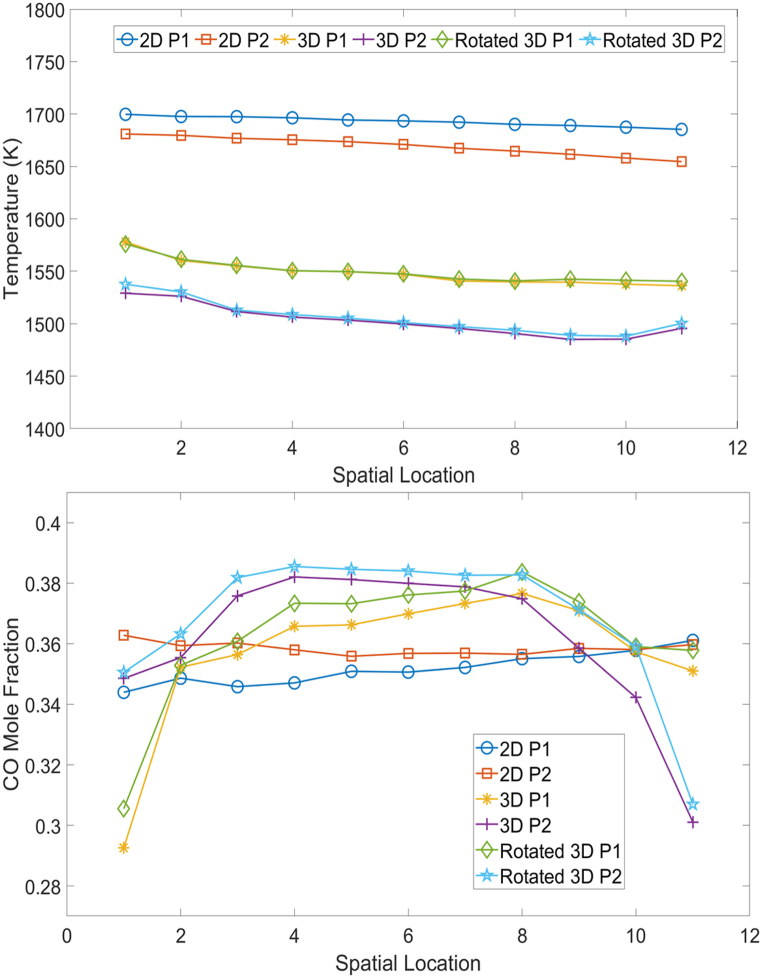
Spatial temperature (top), CO (bottom) distributions from 2D and 3D simulations along particles. P1 represents the front particle, and P2 represents the rear particle in the simulation.

The spatial temperature and CO mole fraction distributions along the particle surfaces are shown in Fig. 5. To facilitate the comparative analysis of spatial data across particles with varying dimensions, a normalization approach was employed using isosurfaces based on one axis. Specifically, for each particle, whether spherical or realistically shaped, the range from the front to tail along the horizontal axis was divided into ten equal intervals with endpoints marked as 1–11 for plotting purposes. At each division point, an isosurface of the particle's cross section was created. It captured only the particle surface, thus facilitating the measurement of averaged variables, such as temperature and species mole fraction. By representing the physical location as ordinal numbers (1, 2, 3, etc.), variables from particles of different sizes were compared directly on the plots, providing a standardized method to assess spatial variations in particle behavior.

For the 2D temperature distribution, both the front particle (2D P1) and rear particle (2D P2) maintained a relatively stable temperature with minor temperature differences along the surface, which contrasted with the varied temperature gradients exhibited by the 3D front particle (3D P1) and rear particle (3D P2). The thermal profile for the 3D simulation was further altered when the front particle was vertically rotated 90° (rotated 3D P1) for both the rotated particle and the subsequent rear particle. This implies that particle orientation plays a crucial role in the heat transfer phenomena during the process.

In the CO mole fraction plot, the difference between the 2D and 3D simulations was more pronounced, suggesting that the complexities of the real particle surfaces and large pores significantly affected the gas-phase species concentrations. The rotation of the front 3D particle caused the reorientation of the reaction sites and flow paths, resulting in marked changes in the CO distribution for both particles. This effect was absent in the 2D simulation, which maintained a more uniform species distribution owing to its simplistic shape.

The influence of particle shape and orientation on the aerodynamic properties of coal and biomass particles in co-firing systems has been analyzed through the examination of drag coefficients in Table 2. From the table, the drag coefficient for the front particle in the 3D model (14.1) was found to be 26.4 % higher than in the 2D model (11.1). This increase is primarily due to the more complex surface and irregular shape of the realistic 3D particle, which creates greater resistance to the flow at low Reynolds number compared to the simplified spherical model used in 2D simulations. Moreover, the rear particle in the 3D model exhibited a drag coefficient of 12.7, which is 53.2 % higher than that of the rear particle in the 2D model (8.3). This substantial increase suggests that the flow disturbances caused by the front particle are more pronounced in the realistic 3D setting, leading to higher drag on the rear particle. The enhanced drag in the 3D scenario underscores the limitations of 2D models, which may significantly underestimate the aerodynamic resistance experienced by particles in practical co-firing systems. Additionally, when the front particle in the 3D model was rotated, the drag coefficient decreased by 16.2 % (from 14.1 to 11.8), while the rear particle's drag coefficient decreased by 5.5 % (from 12.7 to 12.0). This reduction indicates that particle orientation plays a crucial role in determining the flow resistance. The change in orientation alters how the flow interacts with the particle's surface, potentially presenting a less resistant profile and thus reducing the drag. This finding highlights the importance of considering particle orientation in simulations, as it can influence the overall flow dynamics and heat transfer within the system.

**Table 2:** Comparative drag coefficients for front and rear particles in 2D, 3D, and rotated 3D simulations.

	Front Particle (P1)	Rear Particle (P2)
2D Particle	11.1	8.3
Initial 3D Particle	14.1	12.7
Rotated 3D Particle	11.8	12.0

**Fig. 6:**
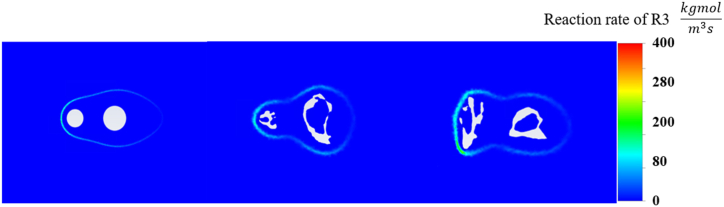
Reaction rate of R3 distributions from 2D and 3D simulations with XY and YZ planes.

Considering the reactions outlined in Table 1, the simulations suggested that the actual morphology and orientation of the particles had a significant impact on the reactions. The uneven surface and nonspherical shape of the real particles appeared to influence the accessibility of the reactants to the particle surface and the removal of products from the surface. This is in contrast to the ideal spherical shape, which exhibited a more uniform and predictable pattern of species distribution. The importance of real particle morphology was further underscored from the perspective of kinetic parameters. The substantial variability in the local surface temperatures and reactant concentrations owing to the complex particle shapes likely affected the local reaction rates, considering their exponential dependence on these factors. As shown in Fig. 6, the reaction rate distribution of R3 is provided where the real particle morphology displayed a varied reaction rate landscape. The highest reaction rate was observed at the location where the reactant was in full contact, such as the front area of the left particle, for both 2D and 3D simulations. Because of the uniform shape, which can result in a more consistent reaction surface, only a small amount of oxygen could be transferred to later particles, causing a lower reaction rate in the 2D simulation. However, real char particles with intense activity in certain regions (coal particles in the XY plane), which made oxygen accessibility unconstrained from the front particle, highlighted the complex interplay between the particle's irregular surface and the reaction process.

**Fig. 7:**
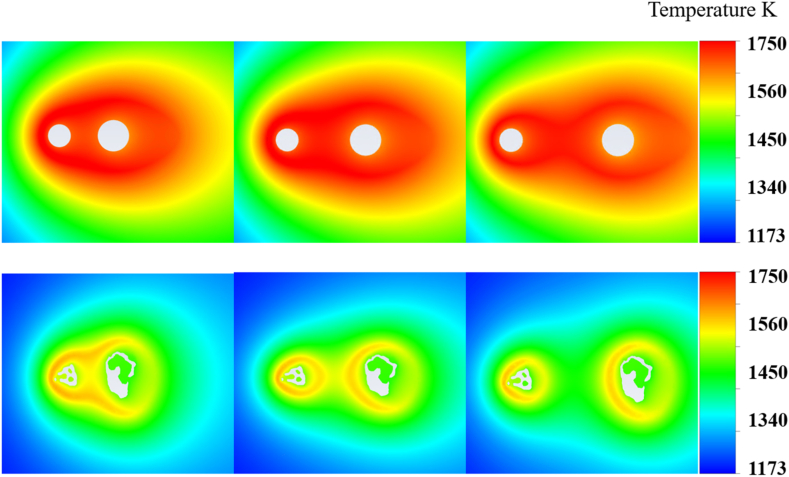
Temperature distributions from 2D (top) and 3D (bottom) simulations with particle distance L (left), 2L (middle) and 3L (right).

The initial setup positioned two particles at a separation distance L, which was defined as half of the average major axis length. To investigate the inter-particle effects under varying conditions, the separation was increased to both 2 L and 3 L. Fig. 7 shows the resulting temperature distributions for these altered distances, with the upper section of the image representing 2D particles and the lower section representing 3D particles. This approach facilitated a comparative analysis of how increased spacing affected the thermal behavior of particles in both two-dimensional and three-dimensional simulation environments.

As the distance between the particles increased, for both the 2D and 3D simulations, the temperature fields became more isolated with increasing particle separation. The temperature decreased from 1676 to 1672 K in the 2D simulations and from 1510 K to 1499 K in the 3D simulations, indicating a reduced influence as the distance between the particles increased. This trend suggested that the ideal structure had less impact on thermal interactions when the particles were spaced further apart. At L, there was significant thermal interaction between the particles, as indicated by the merged high-temperature zones. At 2 L, the interaction decreased, and distinct high-temperature zones around each particle became apparent. At 3 L, the thermal effects of the particles on each other were minimal, with each particle exhibiting an individual high-temperature region. However, unlike the 2D simulations, the change in temperature distribution as the distance between particles varied from 2 L to 3 L could be attributed to the interplay between thermal and reactive effects in the 3D simulations. At a closer proximity of 2 L, the elevated temperature at the rear of the front particle suggested that exothermic reactions from the leading particle surface significantly contributed to the thermal profile of the trailing particle. The combustion process generated hot gases that maintained high temperatures around both the particles because of the reduced space for heat dissipation. As the distance increased to 3 L, the influence of these reactive thermal effects decreased. The larger spatial separation facilitated greater heat dissipation into the surrounding environment, resulting in a noticeable drop in the temperature at the rear of the front particle. This indicates a diminished convective heat transfer from the leading particle to the trailing particle, demonstrating the importance of spatial configuration in dictating thermal feedback within particle systems. Moreover, at 3 L, each particle behaved more independently, with combustion primarily influenced by the local availability of reactants and less by the thermal output of neighboring particles. Thus, the temperature field provides a more accurate reflection of the reactive behavior of individual particles than of the collective influence of closely packed particles.

**Fig. 8:**
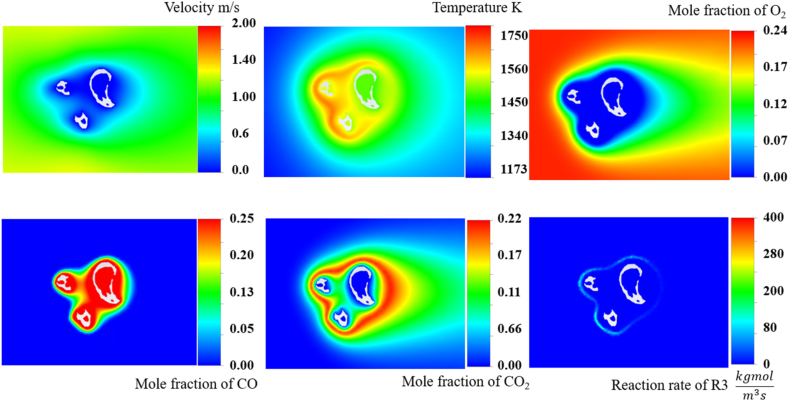
Velocity, species, and reaction rate (R3) distributions for three particles.

As indicated in the previous figures, the ideal structure of the char particles is limited in representing the real reactivity of the reaction process. To increase the complexity of the simulation and make it more representative of actual environments in which many particles are present and interact, Fig. 8 shows the temperature, velocity, species, and reaction rate distributions for the three particles in a combustion environment, which offers a complex scenario for analysis. With the addition of a third particle, the flow dynamics became more intricate, as evidenced by the temperature and velocity fields. The presence of multiple particles significantly altered the flow, creating zones of recirculation and areas wherein the velocity and temperature were markedly decreased or increased, owing to the obstruction and redirection of flow paths.

The distributions of O_2_, CO, and CO_2_ also became more heterogeneous with the introduction of a third particle. The O_2_ concentration decreased in the regions closest to the particles where the reactions were most intense, whereas the CO and CO_2_ concentrations were highest in or around these areas, indicating an active reaction process. The increased complexity of the flow field, owing to the interaction of the irregularly shaped particles, likely contributed to the varied distribution of these species. The reaction rate distribution illustrates the non-uniformity of the reactive sites across the particles. This highlights the importance of considering the real morphology of the char particles in simulations to accurately predict the reaction rates. Further, the interplay between the particle-induced flow disturbances and chemical reactions became more pronounced as the number of particles increased, necessitating the use of sophisticated models to consider these effects. The inclusion of a third particle demonstrated that as the system approached the more realistic particle loading found in actual reactors, the flow and reaction patterns become increasingly complex. However, the number of simulated particles was far less than that in the actual situation, wherein more than hundred thousand particles were transferred. A faster and more representative simulation is needed in future work to understand the interactions at the particle scale.

## 4 Conclusions

This study conducted a preliminary and comprehensive numerical investigation of the interparticle effects among multiple coal char particles during co-firing with biomass. A novel approach that integrated micro-CT-based realistic particle morphology was employed. A comparative analysis of chemical reaction simulations using both idealized and realistic particle morphologies showed significant differences in the behavior of the chemical species and reaction rates during the co-firing of biomass and coal. This study underscores the critical impact of the real particle morphology on the reaction process, with irregular shapes and surfaces leading to more complex interaction patterns that cannot be captured by simplified spherical models. The use of micro-CT imaging to inform CFD simulations represents a substantial advancement in the accuracy and reliability of predictive modeling for co-firing systems.

The findings of this study indicated that the incorporation of realistic particle geometries was essential for capturing the true dynamics of the interparticle effects and reaction mechanisms. This approach has the potential to significantly enhance the efficiency of co-firing processes by providing a deeper understanding of the fundamental processes involved. Future research should involve a larger number of particles with different distances to better understand the inter-particle interactions in practical scenarios. Additionally, incorporating more detailed chemical mechanisms will enhance the accuracy of the simulations to include the effects of particle shape on combustion efficiency and emissions. Although the present study focuses on numerical simulations to demonstrate the impact of realistic particle morphology on temperature distributions and flow dynamics, experimental validation is recognized as a critical next step. Therefore, we also plan to include experimental studies to validate our simulation results and provide a comprehensive comparison, further improving the reliability of our findings. With the findings advocating for more sophisticated simulation techniques, this research lays the groundwork for optimizing the design and operation of co-firing reactors, ultimately contributing to the advancement of sustainable energy solutions.

## Data availability statement

The data associated with this study have not been deposited in a publicly available repository. Data are available from the corresponding author upon request.

## Declaration of generative AI and AI-assisted technologies in the writing process

During the preparation of this work the author used ChatGPT in order to improve readability. After using this tool, the author reviewed and edited the content as needed and take full responsibility for the content of the publication.

## Declaration of competing interest

The authors declare that they have no known competing financial interests or personal relationships that could have appeared to influence the work reported in this paper.
